# Wood Transcriptome Profiling Identifies Critical Pathway Genes of Secondary Wall Biosynthesis and Novel Regulators for Vascular Cambium Development in *Populus*

**DOI:** 10.3390/genes10090690

**Published:** 2019-09-07

**Authors:** Min-Ha Kim, Jin-Seong Cho, Hyung-Woo Jeon, Kanidta Sangsawang, Donghwan Shim, Young-Im Choi, Eung-Jun Park, Hyoshin Lee, Jae-Heung Ko

**Affiliations:** 1 Department of Plant & Environmental New Resources, Kyung Hee University, Yongin 446-701, Korea (M.-H.K.) (J.-S.C.) (H.-W.J.) (K.S.); 2 School of Life and Environmental Sciences, The University of Sydney, Camperdown, NSW 2006, Australia; 3 Korea Forest Research Institute, Suwon 16631, Korea (D.S.) (Y.-I.C.) (E.-J.P.) (H.L.)

**Keywords:** poplar, Ptrham4-1, tissue-specific transcriptome, vascular cambium, wood formation

## Abstract

Wood, the most abundant biomass on Earth, is composed of secondary xylem differentiated from vascular cambium. However, the underlying molecular mechanisms of wood formation remain largely unclear. To gain insight into wood formation, we performed a series of wood-forming tissue-specific transcriptome analyses from a hybrid poplar (*Populus alba* × *P. glandulosa*, clone BH) using RNA-seq. Together with shoot apex and leaf tissue, cambium and xylem tissues were isolated from vertical stem segments representing a gradient of secondary growth developmental stages (i.e., immature, intermediate, and mature stem). In a comparative transcriptome analysis of the ‘developing xylem’ and ‘leaf’ tissue, we could identify critical players catalyzing each biosynthetic step of secondary wall components (e.g., cellulose, xylan, and lignin). Several candidate genes involved in the initiation of vascular cambium formation were found via a co-expression network analysis using abundantly expressed genes in the ‘intermediate stem-derived cambium’ tissue. We found that transgenic Arabidopsis plants overexpressing the *PtrHAM4-1*, a GRAS family transcription factor, resulted in a significant increase of vascular cambium development. This phenotype was successfully reproduced in the transgenic poplars overexpressing the *PtrHAM4-1*. Taken together, our results may serve as a springboard for further research to unravel the molecular mechanism of wood formation, one of the most important biological processes on this planet.

## 1. Introduction

Woody biomass represents more than 90% of the total biomass produced within the earth’s terrestrial ecosystems. Roughly 25% of the annual anthropogenic CO_2_ emissions can be assimilated during woody biomass formation, suggesting that wood formation serves as one of earth’s major long-term terrestrial carbon sinks [1,2]. Woody biomass has the potential to be renewable as well as carbon neutral with regard to its conversion into various forms of energy (e.g., electricity, gas, and liquid energy); thus, it has attracted attention in fields of sustainable energy [3,4,5,6,7,8,9,10].

Wood is primarily produced by woody perennials through a process called secondary growth (i.e., wood formation). Secondary growth is achieved by the vascular cambium, a cylindrical domain of pluripotent stem cells below the organ surface, forming wood (i.e., secondary xylem) inside and bast (i.e., secondary phloem) outside in a strictly bidirectional manner by coordinated cell division and differentiation [11]. However, the underlying molecular mechanisms of vascular cambium initiation/proliferation and vascular patterning remain largely unclear. Since the herbaceous model plant Arabidopsis undergoes secondary growth within the stem, root, and hypocotyl [12,13], most of our current understanding of secondary growth comes from Arabidopsis studies. Recently, the PXY/TDR (PHLOEM INTERCALATED WITH XYLEM/TDIF RECEPTOR) signaling network has been reported [14,15], which is highly conserved among euphyllophytes (i.e., ferns and seed plants) [16]. PXY/TDR binds the CLE (CLV3/EMBRYO SURROUNDING REGION)-related peptide CLE41/CLE44/TDIF and activates the WOX4 and WOX14 transcription factors [17,18]. WOX4, expressed in the cambium, promotes stem cell proliferation by interacting with HAM4 (HAIRY MERISTEM 4), one of four HAMs in Arabidopsis [19]. HAM proteins are members of the GRAS (GAI, RGA, SCR) family of transcription factors and are essential components of a non-cell autonomous signaling pathway for maintenance of shoot meristem identity [20,21]. Additionally, PXY-TDIF signaling reduces Brassinosteroid (BR) signaling associated with xylem differentiation by interacting directly with BIN2 (BRASSINOSTEROID-INSENSITIVE2) [22,23].

Wood comprises of secondary cell walls consisting primarily of three polymers: Cellulose; hemicellulose; and lignin [24]. Cellulose microfibrils provide load-bearing strength to the cell wall by forming scaffolds with other wall polymers, such as xylan and lignin [25]. Xylan is one of the major hemicelluloses found in the secondary cell walls of poplar, and, unlike cellulose, has reducing end oligosaccharides with a variety of side chains [26]. Lignin is a complex phenolic compound providing compression strength and hydrophobicity to secondary walls [4,27]. Biosynthesis of these cell wall components is specifically regulated by multi-layered transcription factor networks [28].

Many large-scale gene expression analyses have been performed to understand these regulatory networks [29,30,31,32,33,34,35,36,37,38]. For example, Yang et al. [33] in their study identified many candidate genes for cell wall biosynthesis using comparative genomics of Arabidopsis and *Populus*. Cai et al. [34] uncovered cell wall-related genes in *Populus* using co-expression network analysis. Taylor-Teeples et al. [35] identified 50 genes for xylem cell specification including transcription factors and enzymes involved in cellulose, hemicellulose, and lignin biosynthesis by systematically mapping the regulatory network at cell-type resolution in Arabidopsis. Shi et al. [36] identified multiple transcription factors and wood component genes within *Populus trichocarpa* using the transcriptomes of five tissues (xylem, phloem, shoot, leaf, and root) and two wood-forming cell types (fibers and vessels). In addition, Sundell et al. [37] reported a high spatial-resolution transcriptome analysis spanning the secondary phloem, vascular cambium, and wood-forming tissues of *Populus tremula*, which were obtained by longitudinal tangential cryosections, and then provided an interactive Web resource capable of exploring expression profiles and co-expression networks, the AspWood (http://aspwood.popgenie.org). Very recently, Chao et al. [38] reported on the developmental dynamics of the *Populus* stem transcriptome to identify differentially-expressed transcripts by PacBio full-length sequencing and RNA-seq analysis that are involved in the transition from primary to secondary growth.

*Populus* is employed as a model system for secondary growth not only due to its inherent massive wood formation but also because it is an important source of biofuel [39,40,41,42]. Within *Populus* secondary growth, it has been reported that PXY-TDIF signaling is conserved [43]. However, secondary growth of woody perennials seems to differ substantially from that of Arabidopsis root or hypocotyl; thus, it is imperative to identify the genes regulating secondary growth in *Populus* as a woody model [28,36,44].

In this report, we analyzed a series of wood-forming tissue-specific transcriptomes from a hybrid poplar to understand the molecular mechanism of xylem cell differentiation from cambial cell fate specification. Cambium and xylem cells from a gradient of secondary growth developmental stages (i.e., immature, intermediate, and mature stem) were isolated and RNA-seq libraries were constructed. We found many critical players catalyzing each secondary wall biosynthetic step as well as novel candidate genes possibly involved in the vascular cambium initiation using a proof-of-concept experiment.

## 2. Materials and Methods

### 2.1. Plant Materials, Growth Conditions, and RNA Sequencing

Hybrid poplars (*Populus alba × P. glandulosa*, clone BH) were grown in a field (latitude 37.2N, longitude 126.9E) after transplanting two-month-old plants grown in a growth room (16 h of light; light intensity, 150 μmol/m^2^/s; 24 °C). This clone was selected and utilized in this study because it is easy to transform the gene of interest for further study. Tissue samples were collected as described previously [45] and combined from at least 10 one-year-old trees. In brief, shoot apical meristem samples were collected from the top of the shoot, and immature, intermediate, and mature stem samples from the second to third internodes, eighth to fifteenth internodes, and twenty-fifth internodes (diameter 1.0–1.5 cm), respectively. Collected leaf samples were from fully expanded young leaves without major veins. Cambium tissue was collected by scraping the inner part of the detached bark using a double edge razor blade, and developing xylem was collected by scraping the surface of the debarked stems. The total RNA was isolated using the cetyl trimethylammonium bromide (CTAB) method with slight modifications [46]. In brief, fine powder from plant tissues was mixed with a CTAB buffer, followed by a phenol/chloroform/isoamyl alcohol (25:24:1) extraction. Isopropanol was added to the mixture to isolate RNA, the RNA quality was estimated using an Agilent 2100 Bioanalyzer (Agilent, Santa Clara, CA, USA). One microgram of total RNA from each sample was used for an RNA sequencing library construction following the Illumina TruSeq RNA library type (Illumina, San Diego, CA, USA). The libraries were quantified using a 2100 Bioanalyzer (Agilent Technologies, Santa Clara, CA, USA) and paired-end sequencing was performed on an Illumina HiSeq2000 platform (Illumina). The resulting RNA sequencing data were deposited in NCBI with BioProject accession (PRJNA522944; https://dataview.ncbi.nlm.nih.gov/object/PRJNA522944?reviewer=56urghn66puds3q2e274rsiuvl). 

### 2.2. Transcript Assembly, Abundance Estimation, and Annotation

Raw reads generated from RNA sequencing were cleaned by PRINSEQ-Lite 0.20.4 (http://prinseq.sourceforge.net/) using a Phred quality score of 20 or above with minimum lengths of 50 bp or greater. For genome-guided transcriptome assembly, a script in Trinity v2.2.0 was used [47,48]. In brief, the cleaned paired-end reads of each library were mapped to the P. trichocarpa genome v3.0 (http://www.phytozome.org) using Bowtie v. 1.2.2 [49]. The transcript abundance (e.g., read count) was determined by RSEM (RNA-Seq by Expectation Maximization, v. 1.3.0) [50] and represented by a FPKM (Fragments Per Kilobase of transcript per Million mapped reads) value. The edgeR was used for statistical analysis of the differential transcript abundance by input read counts [51] using the following script; $run_DE_analysis.pl --counts.matrix --method edgeR --output edgeR_dir --dispersion 0.1 --samples samples.txt. For transcript annotation, BLASTX (e-value 1e-5) was performed against A. thaliana (Athaliana_167_TAIR10) and P. trichocarpa (Ptrichocarpa_210_v3) protein datasets from Phytozome V12 (https://phytozome.jgi.doe.gov/pz/portal.html), respectively.

### 2.3. Quantitative Real-Time PCR (qRT-PCR) and Semi-Quantitative RT-PCR

One microgram of poplar total RNA was reverse transcribed to produce first-strand cDNA using a PrimeScript™ RT reagent kit (Takara, Otsu, Japan) following the manufacturer’s instructions. The gene expression patterns were analyzed by quantitative real-time PCR (qRT-PCR) [52]. All primer sequences were designed using the Primer3 program (http://fokker.wi.mit.edu). Poplar Actin2 gene was used as the quantitative control [53]. The qRT-PCR was performed using a CFX96™ Real-time PCR Detection System (Bio-Rad, Hercules, CA, USA) with iQ™ SYBR^®^ Supermix (Bio-Rad). In Arabidopsis samples, the total RNA was extracted using TRIZOL reagent (Ambion, Austin, TX, USA), according to the suggested protocol, with slight modifications. One microgram of the total RNA was reverse transcribed using Superscript II reverse transcriptase (Invitrogen, Carlsbad, CA, USA) in a 20 μL reaction. RT-PCR was carried out using 1 μL of the two-fold diluted reaction product as a template. Amplified DNA fragments were separated on 1% agarose gel and visualized with ethidium bromide staining.

### 2.4. Vector Construction and Production of Transgenic Plants

The full-length cDNA encoding *PtrHAM4-1* (Potri.005G125800) was amplified from hybrid poplar (clone BH) by PCR and inserted downstream of the 35S promoter in the pK2GW7 vector [54] using the Gateway cloning system in order to produce 35S::PtrHAM4-1 constructs. Vector constructs were introduced into *Agrobacterium tumefaciens* strain C58, which was used to transform *Arabidopsis thaliana* (Columbia; Col-0) and a hybrid poplar by the floral-dip method [55] and leaf disk transformation–regeneration method [56]. All of the constructs used in this study were verified by DNA sequencing. 

### 2.5. Histological Analysis

Rosette level stems of 60-day-old Arabidopsis plants were used to obtain hand-cut cross sections and stained with 0.05% toluidine blue O for 1 min as described previously [56]. Interfascicular cambium-derived tissue (ICD) extensions were measured at all of the interfascicular regions from 60-day-old stem sections immediately above rosette level. In poplar, lengths of cambial layers were measured in a total of eight directions of each stem sections and at least five ramets of each transgenic line (3-month-grown in test tube after subculture) were used for statistical analysis. Images were captured using a microscope (CHB-213; Olympus, Tokyo, Japan) and analyzed by ImageJ software (National Institutes of Health; http://www.nih.gov/). 

## 3. Results

### 3.1. Generation of a Wood-Forming Tissue-Specific Transcriptome from a Hybrid Poplar

**Figure 1:**
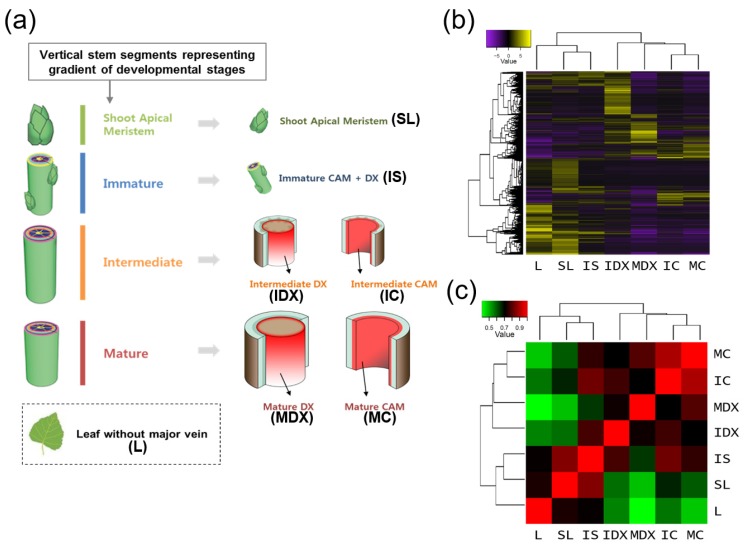
Tissue-specific transcriptome analysis of a hybrid poplar. RNA-seq analysis of various poplar tissues for tissue-specific study. (**a**) Strategy for poplar tissue sampling used in this study. Schematic diagram of tissue samples collected. SL (shoot apical meristem with leaf primordia), IS (immature stem), IC (intermediate cambium), IDX (intermediate developing xylem), MC (mature cambium), MDX (mature developing xylem), and ML (mature leaf without major veins). (**b**,**c**) Sample correlation by heatmap (**b**) and correlation matrix (**c**), which were produced by Trinity (analyze_diff_expr.pl) with log2 fold change value (*p*-value, 0.005).

To explore the molecular mechanism of wood formation, we designed a series of tissue type-specific transcriptome analyses using a hybrid poplar (*Populus alba* × *P. glandulosa*, clone BH). Each wood-forming tissue was isolated from vertical stem segments representing a gradient of developmental stages with regard to secondary growth (i.e., immature, intermediate, and mature stems; Figure 1a), which was previously confirmed by microscopic observation of the stem cross sections [45]. Thus, a total of seven tissue types from at least 10 actively growing poplars (field grown, one-year-old) was collected and combined, including SL (Shoot apical meristem with Leaf primordia), IS (Immature Stem), IC (Intermediate stem-derived Cambium), IDX (Intermediate stem-derived Developing Xylem), MC (Mature stem-derived Cambium), and MDX (Mature stem-derived Developing Xylem). In addition, L (Leaf without major veins) was included as a negative control for wood formation (Figure 1a). SL includes shoot apex with leaf primordia; IS indicates immature stem tissue of the third to the fifth internodes containing procambium and primary xylem; IC and IDX were collected from intermediate stems (seventh to 10th internodes), while MC and MDX were collected from mature stems (20–25th internodes) with a scraping method after separating bark tissues [45] (see Materials and Methods).

The total RNA extracted from the aforementioned tissues was used directly for RNA-seq analysis (Hiseq 2000, Illumina, San Diego, CA, USA). Seven RNA-seq libraries were constructed and sequenced to produce 6.5 to 7.5 million reads per library, corresponding to more than 6 Gb per sample (Supplemental Figure S1). Using genome-guided assembly, about 78.5% of the sequences were mapped to the *Populus trichocarpa* genome (v3.0) (Supplemental Figure S2), with a total of 38,329 transcripts across all tissues after removal of redundant transcripts (from a total of 84,168 transcripts) (Supplemental Table S1).

### 3.2. Reliability of Wood-Forming Tissue-Specific Transcriptome

Transcriptional interrelationships of the seven tissue libraries were evaluated by generating a sample correlation heatmap and matrix using the Trinity package [48] (Figure 1b,c). Interestingly, the seven tissues segregated into two groups: One with L, SL and IS; and the other with ID, MD, IC and MC (Figure 1b). In addition, MC and IC as well as SL and IS were closely located within the same clades. As expected, this result suggests that physiologically similar tissues have comparable transcriptomes. For example, in the correlation matrix corresponding to each tissue, MC is highly correlated with IC (Figure 1c), which is convincing, because both MC and IC consist primarily of cambial cells, and next comes MDX, which flanks MC. Interestingly, the next most correlative tissue to IC is IS-instead of IDX, which flanks IC-implying that IC is related to IS in containing immature meristem tissues. These results suggest that the tissue-specific transcriptome data is reliable.

**Figure 2:**
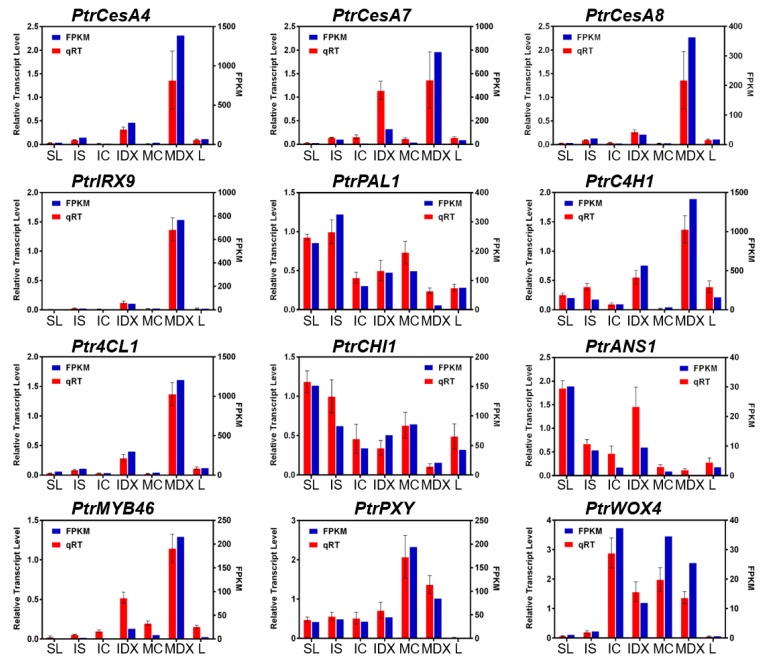
Confirmation of RNA-seq results by independent qRT-PCR analysis. Expression of each indicated gene is plotted. In each plot, blue bars (right Y-axis) indicate FPKM (Fragment Per Kilobase of transcript per Million mapped reads) values from RNA-seq data while red bars (left Y-axis) indicate qRT-PCR results. A gene model name for *P. trichocarpa* (v3.0) is shown in parenthesis as: *PtrCesA4* (Potri.002G257900.1); *PtrCesA7* (Potri.018G103900.1); *PtrCesA8* (Potri.011G069600.1); *PtrIRX9* (Potri.016G086400.1); *PtrPAL1* (Potri.006G126800.1); *PtrC4H1* (Potri.013G157900.1); *Ptr4CL1* (Potri.001G036900.1); *PtrCHI1* (Potri.010G213000.1); PtrANS1 (Potri.003G119100.1); *PtrMYB46* (Potri.009G053900.1); *PtrPXY* (Potri.003G107600.1); and *PtrWOX4* (Potri.014G025300.1). *PtrActin2* (Potri.019G010400.1) was used as a reference gene. Error bars indicate S.E. gene-specific primer sequences used in this analysis are shown in Table S2.

To further verify the RNA-seq data, we performed a real-time quantitative RT-PCR (qRT-PCR) analysis of 12 selected genes, including eight genes involved in secondary cell wall biosynthesis (*CesA4*, *CesA7*, *CesA8*, *IRX9*, *PAL1*, *C4H1*, *4CL3*, *MYB46*), two in anthocyanin biosynthesis (*CHI1*, *ANS1*), and two in vascular cambium development (*PXY*, *WOX4*) (Figure 2). The tissue-specific transcript levels of all the selected genes are very similar to those of the FPKM (Fragments Per Kilobase of transcript per Million mapped reads) values, suggesting that our RNA-seq data with no biological replications is applicable for further in silico analysis as a reference transcriptome.

### 3.3. Identification of Critical Pathway Genes for Secondary Wall Biosynthesis in Populus

**Figure 3:**
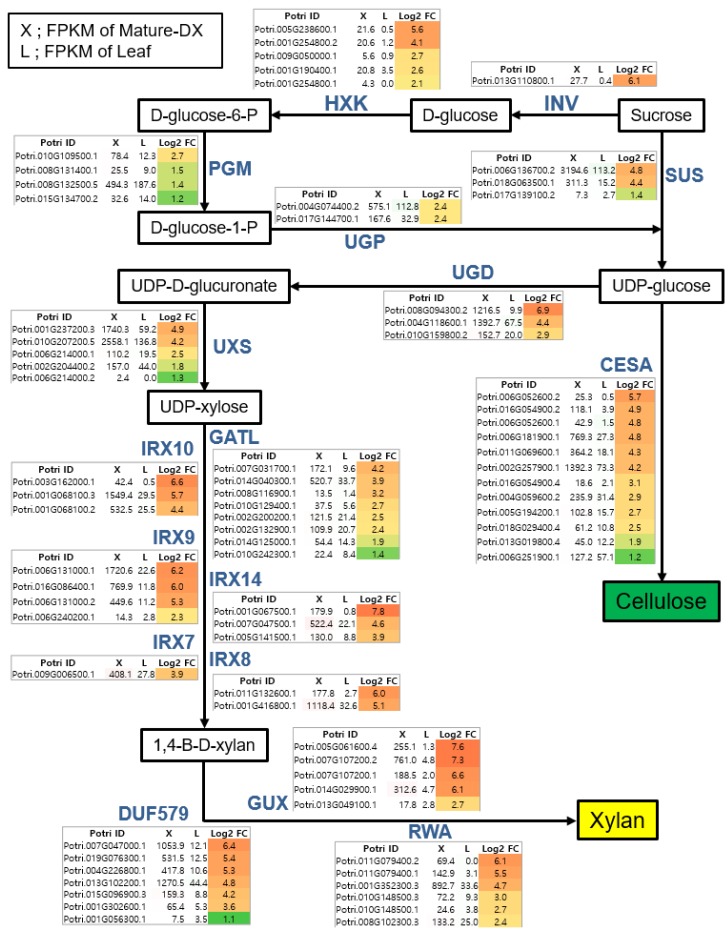
Identification of major players within cellulose and xylan biosynthetic pathways for secondary wall formation. Metabolites in each step of the biosynthetic pathway are shown in the box and all related genes are shown to the side. Fold change (X/L) was calculated in Log2. X is a FPKM value of mature developing xylem (MDX) and L (Leaf without major veins) represents the leaf FPKM value. Color gradient according to the fold change values was visualized.

**Figure 4:**
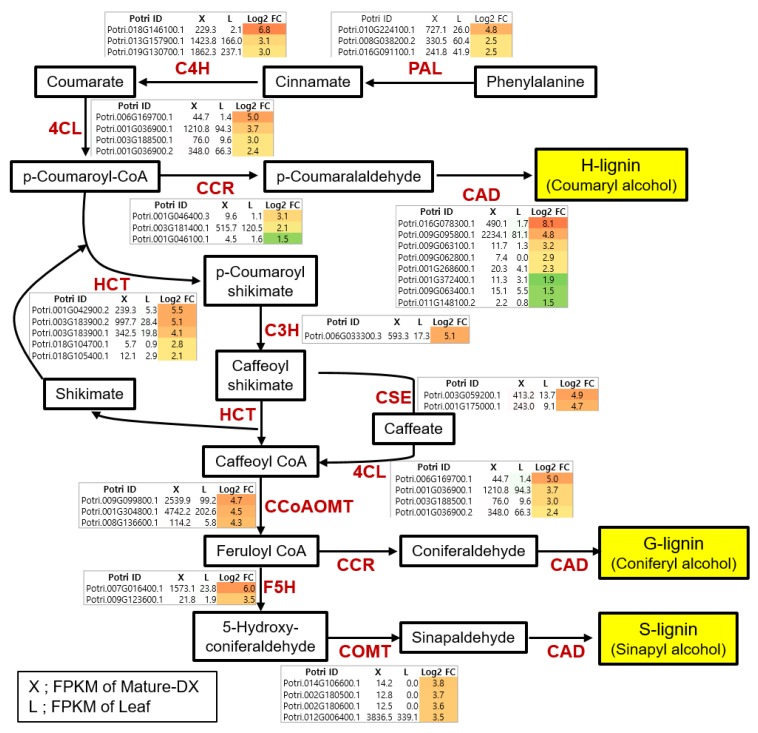
Identification of major players of the monolignol biosynthetic pathway in secondary wall formation. Metabolites in each step of the biosynthetic pathway are shown in the box and all the related genes are shown to the side. Fold changes (X/L) were calculated in Log2. X is a FPKM value of mature developing xylem (MDX) and L is the leaf FPKM value. Color gradient according to fold change values was visualized.

To identify the key players in the biosynthesis pathways of secondary wall components (i.e., cellulose, xylan, and lignin) from a hybrid poplar, we assembled biosynthetic pathways including genes regulating each step, which are upregulated more than two-fold in “MDX” vs. “L” tissue (Figure 3 and Figure 4).

**Figure 5:**
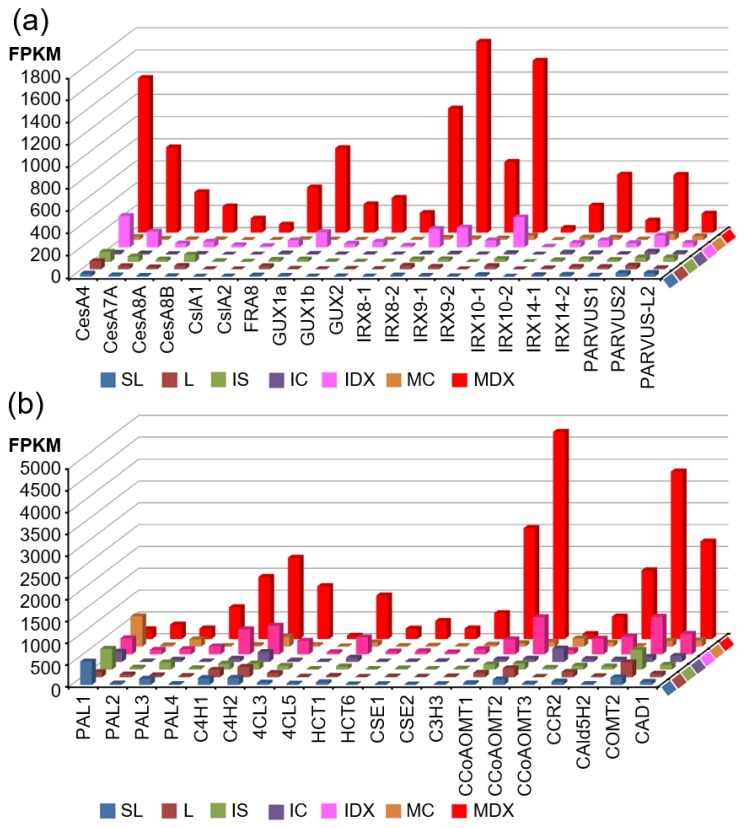
Wood-forming tissue-specific expression of secondary wall biosynthetic genes in poplar. Transcript abundance of all secondary wall biosynthetic genes in each tissue was plotted. (**a**) Genes responsible for biosynthesis of cellulose and hemicellulose: *CesA4* (Potri.002G257900.1); *CesA7-A* (Potri.006G181900.1); *CesA8-A* (Potri.011G069600.1); *CesA8-B* (Potri.004G059600.2); *CslA1* (Potri.008G026400.1); *CslA2* (Potri.010G234100.1); *FRA8* (Potri.009G006500.1); *GUX1a* (Potri.007G107200.2); *GUX1b* (Potri.005G061600.4); *GUX2* (Potri.014G029900.1); *IRX8-1* (Potri.011G132600.1); *IRX8-2* (Potri.001G416800.1); *IRX9-1* (Potri.006G131000.1); *IRX9-2* (Potri.016G086400.1); *IRX10-1* (Potri.001G068100.3); *IRX10-2* (Potri.003G162000.1); *IRX14-1* (Potri.005G141500.1); *IRX14-2* (Potri.007G047500.1); *PARVUS-1* (Potri.002G132900.1); *PARVUS-2* (Potri.014G040300.1); *PARVUS-L-2* (Potri.007G031700.1). (**b**) Genes responsible for monolignol biosynthesis: *PAL1* (Potri.006G126800.1); *PAL2* (Potri.008G038200.2); *PAL3* (Potri.016G091100.1); *PAL4* (Potri.010G224100.1); *C4H1* (Potri.013G157900.1); *C4H2* (Potri.019G130700.1); *4CL3* (Potri.001G036900.1); *4CL5* (Potri.003G188500.1); *HCT1* (Potri.003G183900.2); *HCT6* (Potri.001G042900.2); *CSE1* (Potri.003G059200.1); *CSE2* (Potri.001G175000.1); *C3H3* (Potri.006G033300.3); *CCoAOMT1* (Potri.009G099800.1); *CCoAOMT2* (Potri.001G304800.1); *CCoAOMT3* (Potri.008G136600.1); *CCR2* (Potri.003G181400.1); *CAld5H2* (Potri.007G016400.1); *COMT2* (Potri.012G006400.1); *CAD1* (Potri.009G095800.1). Transcript abundance on the Y-axis represents FPKM values.

Figure 3 illustrates the biosynthetic pathways of cellulose and xylan with sucrose as a starting molecule. The monolignol biosynthetic pathway with phenylalanine as a starting molecule is displayed in Figure 4. For each step in the biosynthetic pathway, the responsible enzymes are mostly encoded by multi-genes. However, based on our transcriptome data, we were able to pinpoint the key players within each step. For example, among the eight genes encoding *CAD (CINNAMYL ALCOHOL DEHYDROGENASE)*, Potri.009G095800.1 is likely the most important due to it being the most highly expressed in terms of quantity (2234.1) in MDX, with >27-fold upregulation compared to L. In addition, Potri.016G078300.1 may be considered an important player, as it exhibited the highest specificity (>228-fold upregulation) in MDX compared with specificity of L, although its quantity was rather low (490.1) (Figure 4). Likewise, of the three genes encoding *SUS* (*SUCROSE SYNTHASE*), Potri.006G136700.2 is likely the principal gene as indicated by its high quantity and specificity in MDX. Combining all the wood-forming tissue-specific expression analyses, we were able to identify key players in each step of the biosynthesis pathways in terms of secondary wall components (Figure 5).

### 3.4. Identification of Genes Involved in Vascular Cambium Initiation and Maintenance

Next, we analyzed our transcriptome data to identify genes that were involved in vascular cambium formation, of which little information is available to date, especially for woody perennials. Firstly, we checked expression of homologs to Arabidopsis genes known to be involved in cambium formation (Table 1). Interestingly, most of these were highly expressed in either IC or MC, or both IC and MC. For example, poplar genes homologous to *PIN4*, *LOG1,* and *AtHAM4* were highly expressed in IC; while *PIN1*, *AHK4/CRE1/WOL1*, *AHP1*, *CLE41*, *CLE44*, *AtAUR1*, *MOL1,* and *PXY* homologs were highly expressed in MC. In contrast, *WOX4*, *TMO5-Like1*, *LHW*, *TMO3/CRF2*, *AtHB8*, *SHR*, *TMO6,* and *DOF5.6* were abundantly expressed in both IC and MC when compared to other tissues. These results suggest that poplar has a gene regulatory network of cambial development similar to that identified in Arabidopsis.

To identify novel genes involved in vascular cambium initiation and maintenance within poplar, we attempted to isolate a group of genes preferentially expressed in either IC or MC. To do so, we selected genes preferentially upregulated (>3-fold) in either IC or MC compared to other tissues, which resulted in identification of 885 and 798 transcripts, respectively (Supplemental Tables S3 and S4). Among these, we initially focused on transcriptional regulators (64 and 58 transcripts) (Table 2 and Table 3). Table 2 shows abundantly expressed transcriptional regulators in IC, including homologs to Arabidopsis genes involved in cytokinin signaling (e.g., *ARR11* and *ARR12*), negative regulation of xylem vessel formation (e.g., *ANAC083/VNI2* and *ANAC104/XND1*), and meristem differentiation (e.g., *PNF*, *WOX1*, *AtHAM3*, and *AtHAM4*). Table 3 shows abundantly expressed transcriptional regulators in MC, including genes related to meristem maintenance (e.g., *KNAT6*), asymmetric stem cell division (e.g., *SCHIZORIZA*), and cell proliferation (e.g., *AINTEGUMENTA*, *AINTEGUMENTA-like 5*, *GRF5*). Additionally, abaxial and adaxial cell patterning-related genes (e.g., *KANADI1*, *Dof5.1*) are listed. Thus, the transcripts listed in Table 2 and Table 3 include many genes known to be involved in vascular cambium initiation and maintenance. These results suggest that this listing is reliable and can be used to identify novel regulators of cambium initiation and maintenance in poplar. For example, seven poplar genes homologous to either Arabidopsis *OBP3* or *OBP4* are listed in Table 2, but their functional significance in cambial development remains unknown. Previously, it was found that flowering and development of cambium are inversely correlated [57,58]. Interestingly, transcription factors involved in the negative regulation of flowering were also found (e.g., *EFM*, *NTL8*, *MYR2*) (Table 2).

**Figure 6:**
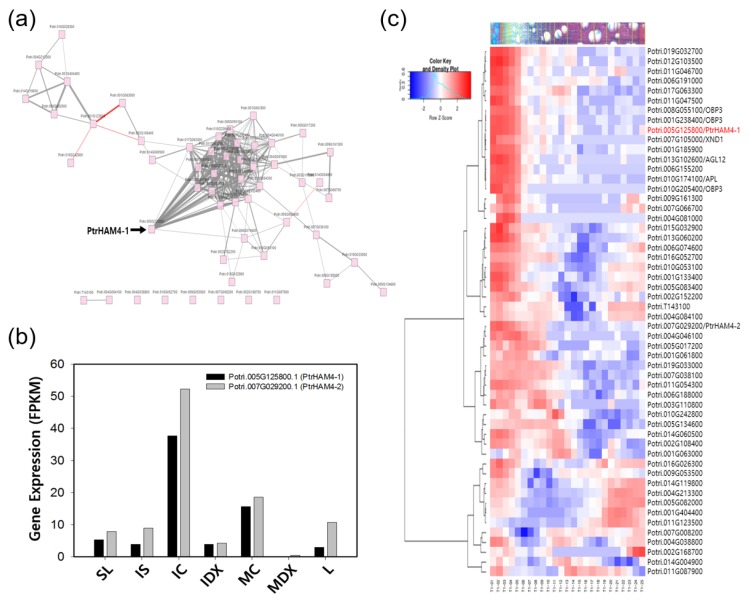
Identification of *PtrHAM4-1*, preferentially expressed in cambium tissue based on in Silico analysis. (**a**) Co-expression network of the intermediate cambium (IC) tissue preferentially expressed transcriptional regulators. The co-expression network was obtained from the AspWood website (http://aspwood.popgenie.org/aspwood-v3.0/) by querying a total of 64 poplar genes (Potri. ID) in the Table 2. *PtrHAM4-1* (Potri.005G125800) was relocated to emphasize. (**b**) Tissue-specific expression of both Potri.005G125800.1/*PtrHAM4-1* and its closest homolog, Potri.007G029200.1/*PtrHAM4-2*. This diagram was reconstructed from our RNAseq data. (**c**) *PtrHAM4-1* is highly expressed in the phloem and cambial tissues. To obtain a gene expression profile by exploiting the high spatial-resolution wood formation data [37] the list of genes from Table 2 was queried to the AspWood website. The resulting heatmap showed that *PtrHAM4-1* (indicated by red letters) is highly expressed in the phloem and cambial tissues.

To discover potential novel regulators involved in the initiation of vascular cambium, we performed a co-expression network analysis using the AspWood website [37] by querying the abundantly expressed transcriptional regulators of IC in Table 2. We found that most of the genes were interconnected with high correlation (Figure 6a). Among them, several genes showed a high degree of prominent centrality based on number of neighbors [37] including Potri.005G125800.1. Both Potri.005G125800.1 and its closest homolog, Potri.007G029200.1, are preferentially expressed in IC (Figure 6b) and show a higher similarity to *AtHAM4* compared to other *AtHAMs*, which were found from both the amino acid sequence alignment (Figure S3a) and phylogenic analysis (Figure S3b). *AtHAM4* was recently reported as a candidate for regulating cambium initiation in Arabidopsis by interacting with WOX4 [19]. Thus, we designated Potri.005G125800.1 and Potri.007G029200.1 as *PtrHAM4-1* and *PtrHAM4-2*, respectively. The high spatial-resolution wood formation data [37] further support their preferential expression in the phloem and cambial tissues (Figure 6c).

### 3.5. Overexpression of Ptrham4-1 Enhanced Vascular Cambium Development in Both Transgenic Arabidopsis and Poplar Plants

**Figure 7:**
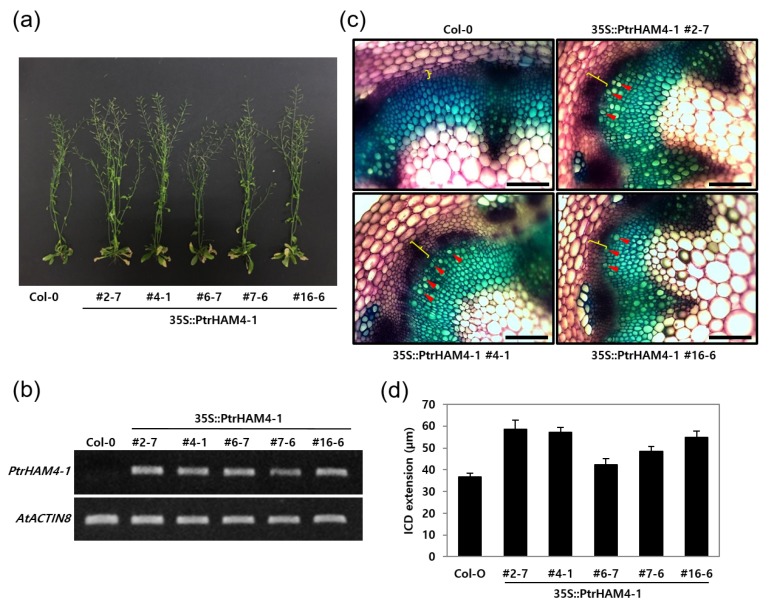
Overexpression of *PtrHAM4-1* in transgenic Arabidopsis increased cambium development. (**a**) Overall growth phenotypes of transgenic Arabidopsis plants. Five independent T3 homozygote transgenic lines (e.g., 2–7, 4–1, 6–7, 7–6, and 16–6) are shown. (**b**) Expression of the *PtrHAM4-1* gene in the independent transgenic Arabidopsis lines compared with Col-0. First-strand cDNA was synthesized from the total RNA extracted from stem tissues and used as a template in semi-quantitative RT-PCR experiments. (**c**) Observation of cambium development in stem cross sections from 60-day-old transgenic Arabidopsis and Col-0. Yellow braces indicate the ICD (interfascicular cambium-derived tissue) extension and red arrowheads point to secondary xylem vessels within the interfascicular region. Scale bars represent 100 µm. (**d**) Quantification of ICD extension in 60-day-old transgenic Arabidopsis plants compared to Col-0. ICD extensions were measured in all interfascicular regions of rosette level stem sections from five independent transgenic lines. Error bars indicate S.E. (*n* = 5).

To test whether *PtrHAM4-1* is functionally involved in cambium formation, we generated transgenic Arabidopsis plants overexpressing *PtrHAM4-1* using the CaMV 35S promoter (i.e., 35S::PtrHAM4-1). Overall, the 35S::PtrHAM4-1 Arabidopsis plants grew normally, but with a dramatic increase of cambial cell proliferation in the inflorescent stems of all five independent T3 homozygous lines (Figure 7). We observed the secondary xylem vessels in the interfascicular region of the 35S::PtrHAM4-1 Arabidopsis plants, which are not found in wild type (WT) plants (Figure 7c). This fact indicates that the vascular cambium was developed in the 35S::PtrHAM4-1 Arabidopsis plants. Accordingly, the 35S::PtrHAM4-1 Arabidopsis exhibited significantly increased interfascicular cambium-derived tissues (ICD) extension of >60% compared to WT plants (Figure 7c,d). 

**Figure 8:**
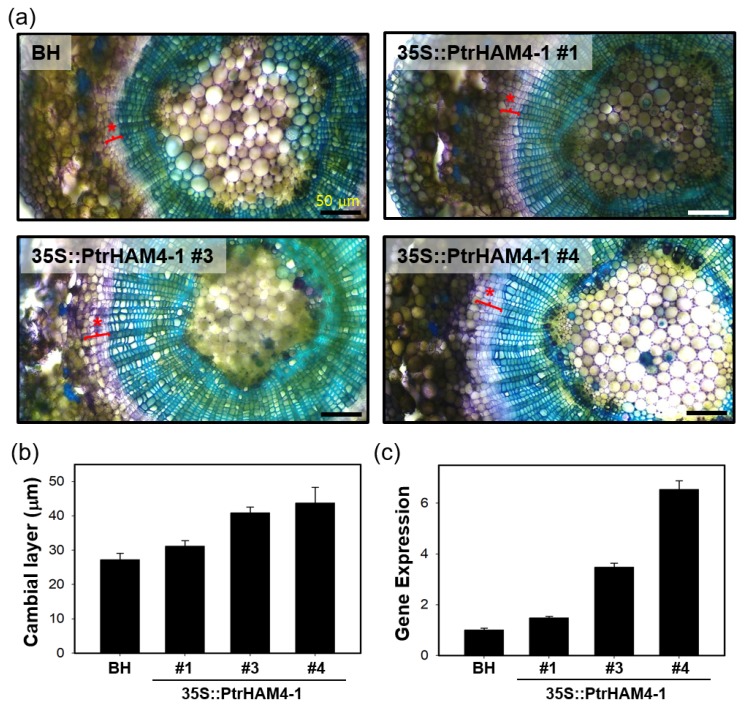
Transgenic poplar overexpressing *PtrHAM4-1* resulted in an increased cambium development. (**a**) Observation of cambium development in stem cross sections of 3-month-old transgenic poplars and wild type BH clone (BH) grown in test tube. Red braces under the red star indicate the cambial layers. Scale bars represent 50 µm. (**b**) Quantification of cambial layers of transgenic poplars compared to BH. The length of cambial layers was measured in stem sections from five ramets of each transgenic lines described in (a). Error bars indicate S.E. (*n* = 5). (**c**) Expression of the *PtrHAM4-1* gene in the independent transgenic poplar lines compared to BH. First-strand cDNA was synthesized from the total RNA extracted from stem tissues and used as a template in the qRT-PCR experiments.

The same construct (35S::PtrHAM4-1) was introduced to a hybrid poplar to further confirm the phenotypic significance of 35S::PtrHAM4-1 Arabidopsis plants. We generated a total of 25 independent transgenic poplar lines and found the increased cambial development (e.g., ICD extension) in many lines compared to the wild-type BH clone (i.e., BH) (Figure 8a). Subsequent quantification and gene expression analysis further showed that the phenotypic significance is nicely correlated with the expression level of the *PtrHAM4-1* gene (Figure 8). Taken together, our results suggest that *PtrHAM4-1* may function as an important player in the initiation of vascular cambium formation in poplar.

## 4. Discussion

Secondary growth is one of the most important biological processes on earth. However, our knowledge concerning the underlying molecular mechanisms of vascular cambium initiation/proliferation and vascular patterning is still fragmented, especially for woody perennials. We hypothesized that genes abundantly and specifically expressed in wood-forming tissues may be important players in wood formation. Recently, many extensive wood-forming tissue-type transcriptome analyses of *Populus* have been reported. Shi et al. [36] in their study used four tissues (shoot tip, leaf, xylem, and phloem) and two wood-forming cell types (fibers and vessels) of *P. trichocarpa*. Sundell et al. [37] presented high spatial-resolution transcriptome data spanning the phloem, vascular cambium, early/developing xylem, and mature xylem with successive longitudinal tangential cryosections of *P. tremula* stem. Chao et al. [38] used three tissues from a hybrid poplar (*Populus deltoids* x *P. euramericana* cv. ‘*Nanlin895*’): Shoot apex; internodes 1–3 (IN1–3); and internodes 4–5 (IN4–5).

Here, we emphasized the transcriptome analysis of cambium differentiation and xylem cell fate specification by designing a gradient of secondary growth developmental stages, including cambium and xylem cell types, within a total of seven tissues: SL; IS; IC; IDX; MC; MDX; and L (Figure 1a). To overcome the limitations of our single run transcriptome data (i.e., no biological replication), we tried; (1) to minimize the biological variation of the samples by combining tissue samples from at least 10 poplars; (2) to check the reliability of our wood-forming tissue-specific transcriptome data by employing the sample correlation heatmap and matrix data (Figure 1c); (3) to validate our transcriptome data using well-known tissue-specific marker genes (Figure 2). We performed not only an extensive transcriptome analysis but also a proof of concept experiment to investigate the molecular mechanism(s) underlying wood formation in poplar.

### 4.1. Critical Pathway Genes of Secondary Wall Biosynthesis in Woody Perennials

Utilizing our series of poplar wood-forming tissue transcriptomes, we sought to uncover the key players for each step in the biosynthesis pathways of secondary wall components (i.e., cellulose, xylan, and lignin) (Figure 3, Figure 4 and Figure 5). Synthesis of secondary wall components depends on the import of sucrose and its subsequent metabolism. However, our understanding of cell wall precursor biosynthesis in developing wood is still limited [24].

Cellulose is a major constituent of plant cell walls and provides load-bearing strength by forming scaffolds with other cell wall polymers, such as xylan and lignin. Arabidopsis has six *SUS (SUCROSE SYNTHASE)* genes; *SUS* is believed to be a main route of carbon entry from sucrose to cellulose via production of UDP-Glc, a substrate for the cellulose synthase [25]. Of the seven *SUS* genes within our transcriptome data, three are highly expressed in MDX compared to expression in L tissue (Figure 3), indicating that these genes are important for cellulose biosynthesis in xylem. Among them, Potri.006G136700, the closest homolog to Arabidopsis *SUS4* (AT3G43190), can be regarded as a key player as it exhibits the highest expression and specificity in MDX. An alternative pathway to production of UDP-Glc from sucrose is through invertases and UGP (UDP-GLC PYROPHOSPHORYLASE). However, UGP is not known to be rate-limiting for cell wall synthesis [59]. We found the gene Potri.013G110800 within the 13 invertases in our data, which is a homolog of Arabidopsis *CINV2 (CYTOSOLIC INVERTASE)* (AT4G09510) with a strong specificity to xylem. It has been reported that loss of cytosolic invertase affects cell wall synthesis in Arabidopsis [60]. Accordingly, Rende et al. [61] suggested that Potri.013G110800 (reported as CIN12) is responsible for supplying UDP-Glc for cellulose biosynthesis in the development of wood of a hybrid aspen (*Populus tremula* × *tremuloides*). Cellulose is produced at the cell surface by CESAs (CELLULOSE SYNTHASE A) [62]. Out of the 13 CESAs in our data, 10 exhibited higher expression in MDX, including: Potri.002G257900.1; Potri.011G069600.1; and Potri.006G181900.1, which are homologous to the well-known secondary wall CESAs; *CESA4* (AT5G44030), *CESA8* (AT4G18780), and *CESA7* (AT5G17420), respectively [63].

Xylans contain xylose subunits as a backbone and are one of the major hemicelluloses found in the secondary cell walls of poplar. Unlike cellulose, xylan has reducing end oligosaccharides, which may act as primers or terminators as well as a variety of side chains [24,26]. UGD (UDP-GLUCOSE DEHYDROGENASE) is responsible for directing xylan biosynthesis from UDP-Glc and for catalyzing the production of UDP-glucuronate [64] (Figure 3). Among the four UGDs within our transcriptome data, three were more highly expressed in MDX—including Potri.008G094300.2—which is highly specific to MDX. UXS (UDP-XYLOSE SYNTHASE) utilizes UDP-glucuronate as a substrate to produce UDP-xylose, a subunit of the xylan backbone. Both Potri.010G207200.5 and Potri.001G237200.3 have higher expression and specificity within MDX, thus, these genes are likely the major UXS genes catalyzing this step. The xylosyl backbone is synthesized by the *IRX9*, *IRX10,* and *IRX14* genes [65]. Our data suggest that Potri.006G131000.1, Potri.001G068100.3, and Potri.001G067500.1 are key players and are homologous to *IRX9*, *IRX10,* and *IRX14*, respectively. In addition, the poplar genes homologous to those in Arabidopsis that synthesize the reducing end oligosaccharide of xylan, such as *IRX7/FRA8*, *IRX8*, and *GATL/PARVUS (GALACTURONOSYL TRANSFERASE-like),* and those responsible for xylan modification, such as GUX, DUF579/GXM, and RWA, are listed in Figure 3. Based on MDX expression and specificity compared to L tissue, we can predict the essential players of each step.

Lignin is a phenolic compound providing compression strength and hydrophobicity to cell walls, and is a highly heterogeneous and complex polymer composed of p-hydroxylphenyl (H), guaiacyl (G), and syringyl (S) units. The lignin monomer biosynthetic pathway has been extensively investigated and well described [27]. In this pathway, 4CL (4-COUMARATE:CoA LIGASE) catalyzes to produce p-coumaroyl-CoA, a precursor for the biosynthesis of all three of the aforementioned monomers. Potri.001G036900.1 (similar to Arabidopsis *4CL2*, AT3G21240.1) seems to be a key gene for this step (Figure 4). S units are synthesized by the action of F5H (FERULATE 5-HYDROXYLASE) and COMT (CAFFEIC ACID O-METHYLTRANSFERASE). Potri.007G016400.1 and Potri.012G006400.1 (similar to Arabidopsis *F5H*, AT4G36220.1 and *COMT*, AT5G54160.1, respectively) are prominent within these steps in poplar (Figure 4). Recently, the CSE (CAFFEOYL SHIKIMATE ESTERASE, AT1G52760.1) enzyme has been added to the lignin pathway of Arabidopsis. CSE provides an alternative route to caffeoyl-CoA by catalyzing caffeoyl shikimate [6]. The poplar genome has two CSEs (Potri.001G175000.1 and Potri.003G059200.1) and both exhibit high expression in MDX (Figure 4). Since Arabidopsis *cse* mutants deposit less lignin but display a four-fold increase in saccharification yield without pretreatment [6], *CSE* is a promising target for the development of improved lignocellulosic biomass. Recently, Saleme et al. [66] reported that silencing *CSE* in a hybrid poplar that is morphologically indistinguishable from WT poplar resulted in up to 25% and 62% reduced lignin deposition and increased glucose release, respectively, without pretreatment.

This analysis was able to identify all the essential genes within each step of the biosynthesis pathways for secondary wall components (Figure 3, Figure 4 and Figure 5), with these genes being potential targets for biotechnological improvement for the purpose of ascertaining quality of woody biomass. Very recently, Wang et al. [67] reported a multi-omics quantitative integrative analysis of lignin biosynthesis by perturbing 21 pathway genes to advance the strategic engineering of wood utilization, and these 21 target genes are exactly matched to key players identified from our analysis, with the exception of CSE (Figure 5b). A similar approach could be applicable to other pathways (i.e., those for cellulose and xylan) in combination in order to produce desirable woody biomass.

### 4.2. Transcriptional Regulators Involved in Vascular Cambium Development

Previously, Pineau et al. [68] reported that the *hca* (high cambium activity) mutant showed premature and numerous cambial cell divisions in both the fascicular and interfascicular regions. To determine the influence of the *hca* mutation on global gene expression they performed transcriptome profiling and found *AtHAM4/SCL15* (At4g36710), the most upregulated transcription factor belonging to a member of the HAM family, which plays an essential role in shoot meristem maintenance in a non-cell-autonomous manner [20,21]. Zhou et al. [19] demonstrated that HAM family members act as conserved interacting cofactors with WUS/WOX (WUSCHEL/WUSCHEL-RELATED HOMEOBOX) proteins. WUS is a homeodomain transcription factor expressed in the rib meristem of the SAM (shoot apical meristem), and is a key regulatory factor controlling stem cell populations in Arabidopsis [69]. WOX4, expressed in Arabidopsis procambial cells, defines the vascular stem cell niche and regulates cambial cell proliferation [15,18,70]. In particular, Zhou et al. [19] showed that HAM4 and WOX4 physically interact in vivo and were tightly co-regulated in both a spatial and temporal manner. For example, *HAM4* and *WOX4* are co-expressed in provascular or procambial cell types of various tissues and, in the stem transverse section, *HAM4* is expressed specifically in the procambium and overlaps with *WOX4* expression. Recently, Kucukoglu et al. [71] suggested that *WOX4*-like genes regulate cambial cell division activity and secondary growth by using PttWOX4a/b RNAi in *Populus* trees.

In our co-expression network analysis (Figure 6a), we found several transcription factors highly expressed in IC tissue. Among them, we focused on *PtrHAM4-1*, a homolog of *AtHAM4*, which is specifically expressed in the cambial tissues (Figure 6). Indeed, overexpression of *PtrHAM4-1* in transgenic Arabidopsis plants resulted in a dramatic increase of the vascular cambium with clear differentiation of secondary xylem vessels, which were known to be produced only from the vascular cambium (Figure 7). This phenotype was further confirmed by overexpressing the *PtrHAM4-1* in hybrid poplars (Figure 8). To our knowledge, this is the first evidence that *PtrHAM4-1* acts positively in vascular cambium development in plants. Taken together with a report from Kucukoglu et al. [71], our results suggest that a HAM4-WOX4 regulatory module may be conserved in *Populus* to achieve cambial meristem initiation and proliferation during secondary growth. However, the underlying molecular regulatory mechanism remains unknown. Chromatin modifications, including histone acetylation, have been implicated in meristem activity. Thus, interaction of *HAM4/SCL15* with *HDA19* (HISTONE DEACETYLASE19) [72] may provide a clue for the regulation of downstream gene expression, which will be a priority for our future studies.

In summary, a comprehensive wood-forming tissue-specific transcriptome analysis from a hybrid poplar successfully pinpointed many essential genes involved in the biosynthetic pathways of secondary wall components. These genes could be focal points for the biotechnological improvement of wood properties within the production of biomaterials and/or biofuels. Furthermore, the transcriptional regulators involved in vascular cambium development were isolated and demonstrated their validity via functional characterization of *PtrHAM4-1* using a heterologous expression. Thus, our results may offer insights for disentangling the complex mechanisms of wood formation, one of the most important biological processes on this planet.

## Figures and Tables

**Table 1:** Expression of genes involved in the cambium initiation and maintenance.

RSEM_ID ^a^	L	IDX	MDX	SL	IS	IC	MC	Potri ID ^b^	% ^c^	AGI ^d^	% ^e^	Description
GG4654|c0_g1_i1	0.4	6.4	4.9	10.1	3.7	5.9	8.2	Potri.002G024700.1	97.4	AT1G19850.1	75.3	MONOPTEROS/Auxin Response Factor 5
GG29733|c1_g1_i1	1.1	1.0	0.0	8.6	6.4	10.5	0.4	Potri.014G146800.5	95.3	AT1G23080.2	66.1	PIN4, Auxin efflux carrier protein
GG28573|c0_g1_i1	0.7	12.1	25.6	1.2	2.4	37.5	34.7	Potri.014G025300.1	99.5	AT1G46480.1	54.5	WOX4, WUSCHEL related homeobox 4
GG36578|c7_g1_i3	0.5	2.7	1.1	0.8	0.3	4.7	4.4	Potri.019G089000.1	97.0	AT1G68810.1	40.0	ABS5/T5L1(TMO5-LIKE1), bHLH protein
GG25890|c1_g1_i1	4.5	17.3	21.8	12.3	24.0	34.6	77.0	Potri.012G047200.2	99.0	AT1G73590.1	73.0	PIN1, Auxin efflux carrier protein
GG19091|c5_g1_i1	0.9	8.8	3.4	4.3	5.6	14.3	17.1	Potri.008G137900.1	98.0	AT2G01830.2	72.1	AHK4/CRE1/WOL, histidine kinase protein
GG22514|c1_g1_i1	0.3	3.2	6.1	6.5	5.6	7.6	13.7	Potri.010G102900.1	98.8	AT2G01830.3	72.2	AHK4/CRE1/WOL, histidine kinase protein
GG2062|c1_g1_i1	14.9	18.4	12.7	57.1	24.2	33.4	46.3	Potri.001G216900.1	98.0	AT2G27230.2	75.3	LHW, transcription factor-related
GG4628|c1_g1_i2	8.0	15.6	7.0	6.9	5.9	56.7	34.6	Potri.002G024000.2	98.7	AT2G28305.1	75.3	LOG1, Lonely Guy1, lysine decarboxylase
GG27071|c0_g1_i1	0.5	8.0	13.3	8.6	6.8	12.8	37.8	Potri.013G028300.1	97.4	AT3G21510.1	66.4	AHP1, histidine-containing phosphotransmitter 1
GG708|c1_g1_i1	1.2	2.8	2.6	1.8	1.5	29.2	44.6	Potri.001G075200.1	96.7	AT3G24770.1	75.3	CLE41, CLAVATA3/ESR-RELATED 41
GG24830|c0_g1_i1	0.0	0.5	0.4	0.1	0.9	9.7	20.8	Potri.011G102400.2	96.6	AT4G13195.1	75.0	CLE44, CLAVATA3/ESR-RELATED 44
GG8362|c3_g1_i1	4.7	3.3	3.0	10.1	10.9	10.0	14.5	Potri.003G136300.1	97.2	AT4G23750.2	75.3	TMO3/CRF2, Target of MONOPTEROS 3
GG16016|c0_g1_i1	1.1	7.1	4.6	7.9	8.6	9.7	18.8	Potri.006G235000.1	98.0	AT4G32830.1	90.4	AtAUR1, ataurora1
GG34739|c4_g1_i1	0.7	43.3	54.9	6.3	17.5	29.6	31.7	Potri.018G045100.2	98.9	AT4G32880.1	82.2	AtHB8, homeobox protein 8
GG12514|c1_g1_i1	2.5	1.6	0.0	2.9	4.3	20.1	8.3	Potri.005G125800.1	98.0	AT4G36710.1	75.3	ATHAM4, GRAS transcription factor
GG16746|c2_g1_i1	10.8	4.2	0.5	8.0	9.0	52.3	18.7	Potri.007G029200.1	98.0	AT4G36710.1	52.7	ATHAM4, GRAS transcription factor
GG17654|c1_g1_i1	0.1	3.5	0.6	4.2	6.5	14.5	22.1	Potri.007G132000.1	98.0	AT4G37650.1	54.5	SHORTROOT, GRAS transcription factor
GG12811|c0_g1_i1	0.4	7.0	0.4	14.1	5.5	18.5	33.2	Potri.005G161500.3	98.0	AT4G40060.1	38.0	AtHB16, homeobox protein 16
GG35388|c0_g1_i1	3.5	7.5	24.9	7.2	9.7	35.1	104.3	Potri.018G113000.1	98.4	AT5G51350.1	57.0	MOL1, Leucine-rich repeat receptor kinase
GG12545|c1_g1_i1	0.3	1.1	2.8	1.3	1.9	21.4	22.4	Potri.005G134200.1	98.0	AT5G60200.1	75.3	TMO6, Target of MONOPTEROS 6
GG8087|c4_g1_i1	1.8	45.5	85.2	35.8	41.3	36.3	194.8	Potri.003G107600.1	98.7	AT5G61480.1	75.3	PXY, Leucine-rich repeat receptor kinase
GG30939|c0_g1_i1	3.2	1.5	0.0	5.9	1.8	9.1	17.7	Potri.015G077100.1	96.8	AT5G62940.1	56.4	DOF5.6/HCA2, Dof-type zinc finger protein
GG26232|c1_g1_i1	2.1	1.4	0.5	3.3	3.0	14.7	20.4	Potri.012G081300.1	96.2	AT5G62940.1	42.7	DOF5.6/HCA2, Dof-type zinc finger protein

^a^, Identifiers obtained from RSEM (Li and Deway, 2011); ^b^, poplar Gene ID from *P.* trichocarpa v3.0 (Phytozome v12.1); ^c^, % identity from blastX against *P.* trichocarpa v3.0; ^d^, Arabidopsis gene ID; ^e^, % identity from blastX against TAIR10.

**Table 2:** Transcriptional regulators preferentially expressed in the intermediate cambium (IC) tissue.

RSEM_ID ^a^	L	IDX	MDX	SL	IS	IC	MC	Potri ID ^b^	% ^c^	AGI ^d^	% ^e^	Description
GG30488|c4_g1_i2	3.7	4.9	3.3	4.0	4.7	**15.7**	8.2	Potri.015G032900.1	97.0	AT1G14685.1	49.5	basic pentacysteine 2
GG11484|c2_g2_i1	0.0	1.3	1.0	0.8	2.6	**9.7**	5.5	Potri.005G017200.1	96.8	AT1G23380.1	75.3	KNAT6. KNOTTED1-like homeobox gene 6
GG585|c4_g1_i1	2.3	7.4	6.9	5.0	5.2	**37.5**	19.2	Potri.001G061800.1	97.9	AT1G28050.1	75.3	B-box type zinc finger protein with CCT domain
GG17054|c0_g1_i2	0.0	0.0	0.0	0.0	1.3	**6.8**	2.3	Potri.007G066700.1	98.0	AT1G31320.1	80.8	LOB domain-containing protein 4
GG25587|c0_g1_i1	0.0	0.0	0.0	0.0	0.0	**16.8**	2.7	Potri.T054700.1	92.0	AT1G43950.1	35.7	auxin response factor 23
GG38344|c0_g1_i1	0.0	0.3	0.7	0.2	0.0	**6.5**	1.9	Potri.T054900.1	95.8	AT1G34390.1	50.0	auxin response factor 22
GG24361|c1_g1_i1	0.0	0.0	0.0	0.0	0.0	**7.5**	0.0	Potri.011G046700.1	95.6	AT1G61110.1	55.4	ANAC025, NAC domain protein 25
GG9929|c0_g1_i1	0.0	0.0	0.0	1.4	0.8	**8.2**	4.1	Potri.004G081000.1	98.4	AT1G65910.1	75.3	ANAC028, NAC domain protein 28
GG9939|c4_g1_i1	7.3	6.7	3.8	4.9	3.5	**34.1**	18.1	Potri.004G084100.2	99.2	AT1G66140.1	75.3	zinc finger protein 4
GG38162|c0_g1_i1	2.9	1.4	0.8	2.1	1.0	**15.9**	7.2	Potri.T143100.1	99.0	AT1G66140.1	39.1	zinc finger protein 4
GG22021|c2_g1_i1	0.8	1.5	2.7	2.2	0.7	**28.0**	11.3	Potri.010G053100.1	98.0	AT1G67710.1	46.5	ARR11, response regulator 11
GG27777|c1_g1_i1	0.7	2.2	1.1	0.1	1.2	**31.5**	8.5	Potri.013G102600.1	96.6	AT1G71692.1	64.2	AGL12, AGAMOUS-like 12
GG23195|c1_g1_i1	7.5	2.8	0.5	4.3	7.2	**52.8**	17.5	Potri.010G174100.1	99.2	AT1G79430.2	60.0	APL, ALTERED PHLOEM DEVELOPMENT
GG20358|c1_g1_i1	9.4	9.7	12.7	4.6	7.6	**49.6**	19.5	Potri.004G010000.1	98.0	AT2G02060.1	75.3	Homeodomain-like superfamily protein
GG15222|c0_g1_i1	0.6	1.0	0.3	1.0	2.0	**15.9**	8.2	Potri.006G155200.1	98.0	AT2G03500.1	51.6	EFM, EARLY FLOWERING MYB PROTEIN
GG15988|c0_g1_i1	0.0	1.1	0.7	0.3	0.0	**5.5**	1.2	Potri.006G188000.1	98.0	AT2G25180.1	53.8	ARR12, response regulator 12
GG21457|c0_g1_i1	2.6	1.0	0.8	5.2	3.8	**19.2**	12.4	Potri.009G161300.1	98.0	AT2G27300.1	56.0	ANAC040/NTL8, NTM1-like 8
GG11094|c2_g1_i1	0.0	19.4	14.2	14.6	12.6	**73.1**	29.3	Potri.004G213300.1	95.6	AT2G27990.1	75.3	PNF, BEL1-like homeodomain 8
GG29464|c1_g1_i1	4.1	14.8	9.7	18.4	9.5	**85.0**	33.9	Potri.014G119800.1	96.4	AT2G34830.1	60.4	WRKY35/MEE24, WRKY transcription factor
GG10107|c0_g1_i1	0.5	2.5	0.0	1.1	1.3	**7.9**	1.1	Potri.004G102600.1	94.7	AT2G37630.1	75.3	AS1/MYB91/PHANTASTICA-like1
GG5942|c1_g1_i1	3.8	8.0	2.2	3.2	2.7	**29.6**	12.5	Potri.002G152200.1	96.0	AT2G45680.1	75.3	TCP9, TCP transcription factor9
GG27378|c2_g1_i1	4.1	0.8	0.0	0.9	4.1	**21.9**	6.6	Potri.013G060200.4	98.6	AT3G04030.3	59.4	MYR2, Homeodomain transcription factor
GG36059|c1_g1_i1	5.8	2.0	0.4	1.0	2.5	**77.0**	10.6	Potri.019G032700.2	96.3	AT3G04030.3	58.5	MYR2, Homeodomain transcription factor
GG36093|c1_g1_i1	0.9	0.4	0.0	0.0	0.4	**5.3**	4.4	Potri.019G036300.1	97.3	AT3G13540.1	37.9	myb domain protein 5
GG3783|c2_g1_i1	1.1	2.3	0.7	0.3	2.1	**12.2**	1.1	Potri.001G404400.1	98.9	AT3G15510.1	75.3	ANAC056, AtNAC2
GG25019|c1_g1_i1	2.4	2.0	0.1	2.1	1.4	**10.4**	0.4	Potri.011G123500.1	98.1	AT3G15510.1	53.5	ANAC056, AtNAC2
GG32655|c7_g1_i2	3.6	4.5	3.2	1.7	2.4	**16.6**	7.4	Potri.015G039100.1	48.5	AT3G18010.1	39.2	WOX1, WUSCHEL related homeobox 1
GG35905|c0_g2_i1	0.0	2.0	1.9	0.8	3.3	**11.8**	11.5	Potri.T050600.1	100.0	AT3G23240.1	51.3	ethylene response factor 1
GG24276|c7_g1_i1	0.6	2.8	0.0	0.5	1.7	**8.6**	4.4	Potri.016G026300.1	53.9	AT3G42170.1	24.6	DAYSLEEPER, BED zinc finger, HAT family
GG2779|c0_g1_i2	0.0	1.2	1.4	1.2	2.4	**9.7**	3.9	Potri.001G295700.1	96.6	AT3G49940.1	75.3	LOB domain-containing protein 38
GG2261|c1_g1_i1	0.9	0.4	0.7	3.9	3.2	**18.3**	8.9	Potri.001G238400.2	97.6	AT3G55370.1	75.3	OBP3/OBF-binding protein 3, Dof family
GG18276|c1_g1_i1	1.4	0.9	1.0	4.9	4.6	**16.5**	10.8	Potri.008G055100.2	98.0	AT3G55370.2	42.1	OBP3/OBF-binding protein 3, Dof family
GG23518|c0_g1_i1	0.4	1.5	0.5	0.8	1.4	**6.4**	1.8	Potri.010G205400.2	98.2	AT3G55370.2	54.5	OBP3/OBF-binding protein 3, Dof family
GG32069|c2_g1_i1	3.1	5.9	3.8	1.8	1.4	**31.6**	25.1	Potri.016G052700.1	96.8	AT3G57670.1	60.6	NTT, C2H2 zinc finger protein
GG8104|c1_g1_i1	2.6	0.5	0.2	4.1	3.7	**14.7**	1.8	Potri.003G110800.1	97.6	AT4G00150.1	75.3	ATHAM3, GRAS transcription factor
GG28884|c1_g2_i1	14.8	3.5	2.9	6.8	7.5	**78.0**	21.7	Potri.014G060500.2	97.3	AT4G00150.1	62.7	ATHAM3, GRAS transcription factor
GG12113|c0_g1_i1	0.4	2.8	0.9	0.9	5.2	**100.2**	19.3	Potri.005G083400.1	98.0	AT4G14540.1	75.3	NF-YB3, nuclear factor Y, subunit B3
GG594|c0_g1_i2	0.2	3.6	2.8	1.0	1.8	**10.8**	5.9	Potri.001G063000.1	96.6	AT4G20970.1	75.3	basic helix-loop-helix (bHLH) protein
GG28018|c0_g2_i1	29.8	34.3	13.4	22.1	44.2	**167.8**	32.9	Potri.013G129800.1	95.0	AT4G20970.1	37.6	basic helix-loop-helix (bHLH) protein
GG24134|c0_g2_i1	0.0	0.0	0.0	0.0	0.2	**10.4**	1.7	Potri.002G168700.1	34.2	AT4G23810.1	75.3	WRKY53, WRKY transcription factor
GG24745|c5_g1_i4	4.5	2.7	0.4	1.9	4.2	**14.0**	4.6	Potri.011G087900.3	96.3	AT4G26640.2	57.6	WRKY20, WRKY transcription factor
GG1779|c1_g1_i4	3.9	1.4	0.3	1.2	2.9	**32.4**	3.7	Potri.001G185900.3	98.0	AT4G29100.1	75.3	basic helix-loop-helix (bHLH) protein
GG36060|c1_g1_i1	1.1	9.8	4.6	10.4	4.8	**52.2**	45.6	Potri.019G033000.1	94.9	AT4G32890.1	39.6	GATA transcription factor 9
GG12514|c1_g1_i1	2.5	1.6	0.0	2.9	4.3	**20.1**	8.3	Potri.005G125800.1	98.0	AT4G36710.1	53.0	ATHAM4, GRAS transcription factor
GG16746|c2_g1_i1	10.8	4.2	0.5	8.0	9.0	**52.3**	18.7	Potri.007G029200.1	98.0	AT4G36710.1	52.7	ATHAM4, GRAS transcription factor
GG12582|c1_g1_i4	0.0	0.6	0.3	1.1	1.8	**6.4**	0.5	Potri.005G134600.1	98.0	AT4G37180.1	75.3	Homeodomain-like superfamily protein
GG12688|c0_g1_i1	0.0	0.7	1.1	2.1	2.4	**22.4**	6.1	Potri.005G147100.2	98.0	AT4G37790.1	75.3	homeobox protein HAT22
GG16541|c0_g1_i2	1.5	1.6	1.4	0.4	0.6	**7.3**	0.5	Potri.007G008200.1	98.0	AT4G37790.1	63.6	homeobox protein HAT22
GG24113|c2_g3_i1	9.1	9.8	5.7	9.1	23.3	**159.4**	37.1	Potri.010G242800.5	53.6	AT5G04840.1	50.0	bZIP protein
GG15591|c1_g1_i3	2.5	1.1	0.1	0.2	1.1	**23.9**	1.9	Potri.006G191000.2	98.0	AT5G06800.1	52.6	myb-like HTH transcriptional regulator
GG20492|c0_g1_i1	4.3	5.1	3.5	0.8	2.6	**26.1**	11.3	Potri.009G053500.1	98.0	AT5G12850.1	47.4	CCCH-type zinc finger protein
GG26437|c1_g1_i1	0.0	0.2	0.5	3.2	3.6	**16.2**	2.4	Potri.012G103500.2	97.5	AT5G13180.1	48.6	ANAC083/VNI2, NAC domain protein 83
GG33423|c0_g1_i1	22.5	23.1	3.6	19.6	29.7	**91.9**	21.4	Potri.017G063300.1	96.4	AT5G13180.1	41.6	ANAC083/VNI2, NAC domain protein 83
GG1261|c0_g1_i1	2.0	0.7	1.5	1.1	0.0	**13.9**	3.5	Potri.001G133400.1	98.0	AT5G45580.1	75.3	Homeodomain-like superfamily protein
GG14451|c0_g2_i1	1.3	0.0	0.0	1.2	0.0	**5.6**	0.5	Potri.006G074600.1	98.0	AT5G57150.1	48.7	basic helix-loop-helix (bHLH) protein
GG9514|c0_g1_i2	1.3	2.4	4.4	2.6	2.4	**20.2**	7.8	Potri.004G038800.1	97.1	AT5G60850.1	75.3	OBP4/OBF-binding protein 4, Dof family
GG9580|c1_g1_i1	0.3	1.6	1.2	3.7	1.5	**15.8**	6.8	Potri.004G046100.1	97.8	AT5G60850.1	75.3	OBP4/OBF-binding protein 4, Dof family
GG24368|c0_g1_i1	10.4	3.1	0.3	10.5	10.8	**115.5**	10.3	Potri.011G047500.1	97.1	AT5G60850.1	64.7	OBP4/OBF-binding protein 4, Dof family
GG24425|c2_g1_i1	1.7	1.6	2.9	3.1	2.9	**34.4**	20.3	Potri.011G054300.1	97.2	AT5G60850.1	67.9	OBP4/OBF-binding protein 4, Dof family
GG17394|c0_g1_i1	0.1	3.3	1.5	1.5	1.9	**24.6**	8.0	Potri.007G105000.1	98.0	AT5G64530.1	54.6	ANAC104/XND1, NAC domain protein 104
GG12096|c0_g1_i1	1.2	12.3	0.7	6.7	11.0	**51.3**	15.5	Potri.005G082000.5	98.0	AT5G65210.1	75.3	TGA1, bZIP transcription factor
GG16813|c0_g1_i1	3.0	10.2	15.5	6.6	17.3	**79.8**	77.9	Potri.007G038100.1	98.0	AT5G65590.1	58.7	SCAP1/STOMATAL CARPENTER1, Dof family
GG5524|c2_g1_i1	0.8	12.1	6.4	11.9	8.1	**63.1**	41.3	Potri.002G108400.1	98.8	AT5G65640.1	75.3	bHLH93, basic helix-loop-helix protein 93
GG4560|c1_g1_i1	0.0	1.4	1.7	0.0	0.0	**12.9**	1.5	Potri.014G004900.4	37.5	AT5G67580.1	56.7	telomeric DNA binding protein

^a^, Identifiers obtained from RSEM (Li and Deway, 2011); ^b^, poplar gene ID from *P.* trichocarpa v3.0 (Phytozome v12.1); ^c^, % identity from blastX against *P.* trichocarpa v3.0; ^d^, Arabidopsis gene ID, ^e^, % identity from blastX against TAIR10. Bold typefaces emphasize the IC (Intermediate Cambium) value.

**Table 3:** Transcriptional regulators preferentially expressed in the mature cambium (MC) tissue.

RSEM_ID ^a^	L	IDX	MDX	SL	IS	IC	MC	Potri ID ^b^	% ^c^	AGI ^d^	% ^e^	Description
GG5056|c1_g1_i1	0.0	0.6	0.7	0.0	0.0	0.6	**6.5**	Potri.002G009700.2	35.7	AT1G20640.1	75.3	NLP4, NIN-like protein 4
GG11457|c0_g2_i1	0.0	0.0	0.0	1.3	0.0	0.0	**5.5**	Potri.005G014200.1	96.3	AT1G23380.1	75.3	KNAT6, KNOTTED1-like homeobox gene 6
GG1392|c4_g1_i1	0.5	1.4	11.4	1.5	1.0	5.9	**35.5**	Potri.012G087100.1	98.0	AT1G23380.1	53.4	KNAT6, KNOTTED1-like homeobox gene 6
GG5866|c1_g1_i2	0.8	1.0	1.0	1.3	0.5	1.8	**5.4**	Potri.002G142400.1	97.7	AT1G27360.1	75.3	SPL11, squamosa promoter-binding protein like
GG12249|c1_g1_i1	0.6	0.4	0.4	0.8	1.2	6.6	**16.5**	Potri.005G097800.1	98.0	AT1G31320.1	75.3	LOB domain-containing protein 4
GG1322|c3_g1_i2	0.5	0.3	0.2	0.9	0.7	2.9	**6.8**	Potri.001G137600.1	98.0	AT1G32240.1	75.3	KANADI 2, Homeodomain-like protein
GG7970|c2_g1_i2	0.0	2.7	1.1	1.2	0.3	5.8	**14.4**	Potri.003G096300.1	97.4	AT1G32240.1	75.3	KANADI 2, Homeodomain-like protein
GG2584|c1_g1_i1	0.1	1.1	2.9	0.9	1.5	3.2	**17.7**	Potri.001G273700.1	98.3	AT1G46264.1	75.3	Heat Shock TF B4, SCHIZORIZA
GG5686|c0_g1_i1	0.2	1.9	3.5	8.2	2.7	3.0	**30.4**	Potri.002G124800.1	98.9	AT1G46264.1	75.3	Heat Shock TF B4, SCHIZORIZA
GG28582|c0_g1_i1	0.4	1.1	3.5	5.5	2.0	3.2	**28.6**	Potri.014G027100.1	99.5	AT1G46264.1	55.3	Heat Shock TF B4, SCHIZORIZA
GG34793|c2_g1_i2	1.1	2.1	3.0	3.1	0.7	8.3	**11.8**	Potri.018G052200.1	97.9	AT1G55110.1	55.3	AtIDD7, indeterminate (ID)-domain 7
GG10199|c7_g3_i1	1.6	1.4	2.2	1.9	0.8	3.0	**7.1**	Potri.004G081000.1	34.2	AT1G65910.1	75.3	ANAC028, NAC domain protein 28
GG22519|c1_g1_i1	3.7	5.7	6.2	11.2	5.0	19.6	**36.0**	Potri.010G102700.1	95.8	AT1G66140.1	49.7	zinc finger protein 4
GG11684|c1_g1_i1	0.5	5.3	21.4	9.2	3.6	22.9	**77.7**	Potri.005G039800.1	99.6	AT1G72210.1	75.3	bHLH96, basic helix-loop-helix (bHLH) protein
GG27052|c1_g1_i1	0.0	0.9	8.7	1.7	1.0	7.3	**29.9**	Potri.013G025900.1	98.6	AT1G72210.1	47.0	bHLH96, basic helix-loop-helix (bHLH) protein
GG15139|c6_g2_i2	0.0	0.0	1.8	0.0	0.0	1.6	**9.0**	Potri.006G145100.4	98.0	AT1G72830.1	40.8	NF-YA3, nuclear factor Y, subunit A3
GG4774|c0_g1_i1	0.9	2.7	0.0	0.0	0.0	25.6	**35.1**	Potri.002G035000.1	97.9	AT1G75250.2	75.3	RAD-like 6
GG4733|c8_g1_i3	0.0	0.4	0.6	2.8	2.6	3.8	**16.4**	Potri.002G030900.1	98.7	AT1G75430.1	75.3	BLH11, BEL1-like homeodomain 11
GG6382|c2_g1_i1	1.5	0.0	1.6	2.1	1.2	2.6	**7.3**	Potri.006G126300.7	41.7	AT2G37025.1	49.3	TRF-like 8
GG797|c0_g1_i1	0.0	7.1	12.8	2.1	3.5	86.3	**102.5**	Potri.001G083700.1	94.4	AT2G46680.1	75.3	homeobox 7
GG3069|c5_g1_i1	2.1	7.3	8.6	4.6	1.1	11.9	**27.6**	Potri.017G107500.2	50.0	AT3G02380.1	60.4	COL2, CONSTANS-like 2
GG36688|c0_g2_i1	1.2	1.2	0.0	0.0	1.0	7.1	**7.3**	Potri.010G143500.1	27.9	AT3G03450.1	29.8	RGL2, RGA-like 2, GRAS family
GG2395|c0_g1_i1	0.8	0.0	0.9	0.0	0.7	6.1	**13.9**	Potri.016G122500.1	38.5	AT3G03450.1	24.8	RGL2, RGA-like 2, GRAS family
GG11822|c1_g1_i1	1.7	7.3	6.8	4.2	3.3	16.7	**26.4**	Potri.005G055300.1	99.1	AT3G04670.1	75.3	WRKY39, WRKY DNA-binding protein 39
GG27332|c0_g1_i1	0.0	0.8	1.4	3.2	1.4	3.4	**11.4**	Potri.013G056400.1	91.4	AT3G13540.1	56.5	myb domain protein 5
GG34915|c2_g1_i1	0.0	1.2	2.7	2.3	0.4	1.4	**8.9**	Potri.018G065400.1	95.4	AT3G13960.1	25.9	GRF5, growth-regulating factor 5
GG25802|c0_g1_i1	0.5	0.2	0.3	1.4	1.7	7.1	**9.9**	Potri.012G038100.1	99.2	AT3G17730.1	78.1	ANAC057, NAC domain protein 57
GG3512|c3_g1_i1	1.1	1.5	1.6	0.7	0.7	2.5	**5.6**	Potri.001G373300.2	98.7	AT3G18380.1	75.3	DTF2, DNA-binding transcription factor2
GG23364|c0_g1_i1	0.0	2.7	3.2	0.9	1.2	5.6	**19.0**	Potri.010G191500.1	95.5	AT3G18990.1	34.5	VRN1, REDUCED VERNALIZATION RESPONSE1
GG13987|c1_g1_i1	3.5	5.2	3.8	4.2	1.1	13.5	**23.6**	Potri.001G299300.2	98.0	AT3G19500.1	75.3	basic helix-loop-helix (bHLH) protein
GG16812|c2_g1_i1	0.1	18.2	30.2	11.1	14.1	42.1	**135.6**	Potri.007G036400.1	98.0	AT3G50410.1	54.1	OBP1, OBF binding protein 1
GG12474|c0_g1_i1	0.7	0.5	0.5	1.9	0.8	5.1	**9.0**	Potri.005G122700.1	98.0	AT3G50870.1	75.3	GATA18/MNP/HAN, GATA family
GG17903|c0_g1_i1	0.7	7.6	6.6	1.3	2.0	7.5	**27.6**	Potri.008G011900.2	98.0	AT3G54320.1	60.2	WRI1, WRINKLED 1
GG27921|c0_g1_i1	0.7	1.3	3.7	3.0	0.7	8.4	**12.9**	Potri.013G117600.1	96.3	AT3G56770.1	40.1	basic helix-loop-helix (bHLH) protein
GG9055|c0_g1_i1	0.2	0.8	0.5	1.1	0.6	9.8	**17.6**	Potri.014G114300.2	35.9	AT3G60030.1	57.5	SPL12, squamosa promoter-binding protein like
GG6170|c1_g2_i1	0.4	0.5	2.4	1.0	1.2	5.2	**9.9**	Potri.002G174300.1	99.6	AT3G61850.4	75.3	DAG1, Dof affecting germination1
GG30346|c1_g1_i5	0.6	0.7	1.4	0.2	0.2	2.3	**5.4**	Potri.014G075200.1	23.4	AT4G00730.1	68.4	ANL2, ANTHOCYANINLESS 2
GG594|c0_g1_i1	0.6	2.9	8.2	3.7	5.7	26.9	**28.9**	Potri.001G063000.1	97.3	AT4G20970.1	75.3	basic helix-loop-helix (bHLH) protein
GG24745|c5_g1_i6	1.4	2.6	2.1	2.5	2.1	6.5	**7.8**	Potri.011G087900.1	90.9	AT4G26640.2	57.6	WRKY20, WRKY family protein
GG28635|c2_g2_i1	0.9	3.4	1.8	1.7	1.1	3.7	**10.2**	Potri.014G036900.1	81.2	AT4G33280.1	41.1	AP2/B3-like family protein
GG28579|c0_g2_i3	0.0	0.9	1.0	0.0	0.0	2.8	**6.8**	Potri.014G025800.1	93.1	AT4G36930.1	50.7	SPATULA, basic helix-loop-helix (bHLH) protein
GG5586|c3_g1_i2	0.7	1.5	0.0	0.0	0.0	2.6	**8.7**	Potri.002G114800.1	96.5	AT4G37750.1	75.3	AINTEGUMENTA, AP2/ERF protein
GG16531|c0_g2_i1	0.1	1.9	1.4	2.8	2.3	5.3	**9.4**	Potri.007G007400.1	98.0	AT4G37750.1	45.0	AINTEGUMENTA, AP2/ERF protein
GG28425|c2_g1_i1	0.3	0.8	0.6	2.2	1.5	5.3	**7.5**	Potri.014G008100.1	98.6	AT4G37750.1	53.2	AINTEGUMENTA, AP2/ERF protein
GG32690|c0_g1_i1	0.0	0.2	0.8	0.1	0.0	2.1	**6.4**	Potri.016G120800.1	98.2	AT5G01310.1	67.5	APRATAXIN-like
GG14540|c1_g1_i1	1.1	0.7	0.3	3.1	3.9	6.2	**11.9**	Potri.006G084200.1	98.0	AT5G02460.1	43.4	Dof5.1, Dof zinc finger protein
GG7630|c0_g1_i1	0.0	0.6	2.4	1.3	0.3	3.3	**9.7**	Potri.003G064600.1	97.5	AT5G14750.1	75.3	MYB66/WER
GG436|c0_g1_i1	0.6	0.7	2.0	1.3	2.3	7.2	**9.2**	Potri.001G048200.1	98.6	AT5G25190.1	75.3	ESE3, ethylene and salt inducible3
GG34721|c0_g1_i1	0.0	0.6	0.5	0.0	0.5	6.2	**6.5**	Potri.018G044900.2	98.5	AT5G25830.1	49.5	GATA transcription factor 12
GG15888|c0_g1_i1	0.0	3.3	1.8	2.9	4.1	3.9	**17.0**	Potri.006G221500.1	98.0	AT5G35550.1	52.1	MYB123/TT2, TRANSPARENT TESTA2
GG4851|c0_g1_i1	0.0	2.8	8.2	3.5	0.5	4.4	**26.1**	Potri.002G043300.1	98.1	AT5G44210.1	75.3	AP2/ERF protein, ERF9
GG7893|c3_g1_i2	0.0	3.4	6.2	1.1	0.8	6.6	**27.6**	Potri.003G089800.1	97.7	AT5G46590.1	75.3	ANAC096, NAC domain protein 96
GG5152|c2_g1_i1	0.0	0.0	0.9	2.3	4.2	6.3	**21.2**	Potri.002G198100.1	29.6	AT5G49330.1	75.3	MYB111, myb domain protein 111
GG25652|c4_g1_i1	1.3	2.4	2.1	1.3	0.5	5.4	**10.2**	Potri.012G100700.3	36.8	AT5G50670.1	45.1	SPL13B, squamosa promoter-binding protein like
GG14378|c1_g1_i2	0.0	0.9	0.7	0.5	0.6	1.5	**16.4**	Potri.006G066400.1	98.0	AT5G52600.1	49.3	MYB82, myb domain protein 82
GG15367|c3_g1_i3	0.0	3.3	5.7	7.3	1.9	13.7	**34.2**	Potri.006G167700.1	98.0	AT5G57390.1	54.5	AINTEGUMENTA-like 5
GG35176|c0_g2_i1	0.0	1.4	8.8	5.0	6.0	20.0	**64.1**	Potri.018G091600.1	97.6	AT5G57390.1	53.6	AINTEGUMENTA-like 5
GG26499|c3_g2_i1	0.0	0.0	4.6	0.2	0.0	0.6	**14.0**	Potri.012G108500.1	96.1	AT5G61890.1	70.0	EBE, AP2/ERF protein

^a^, Identifiers obtained from RSEM (Li and Deway, 2011); ^b^, poplar gene ID from *P.* trichocarpa v3.0 (Phytozome v12.1); ^c^, % identity from blastX against *P.* trichocarpa v3.0; ^d^, Arabidopsis gene ID, ^e^, % identity from blastX against TAIR10. Bold typefaces emphasize the MC (Mature Cambium) value.

## Supplementary Materials

The following are available online at https://www.mdpi.com/2073-4425/10/9/690/s1, Figure S1: RNA-seq raw data processing, Figure: S2. Mapping of the sequence reads to the genome of *Populus trichocarpa*, Figure S3: *PtrHAM4-1* is homologous to *AtHAM4*, Table S1: A total of 38,330 transcripts across all tissues, after removing redundant transcripts from a total of 85,209 transcripts, Table S2: List of primers used in this study, Table S3: List of transcripts preferentially expressed in IC (885 transcripts), Table S4: List of transcripts preferentially expressed in MC (798 transcripts).
