# Supplementary material for: Wood Transcriptome Profiling Identifies Critical Pathway Genes of Secondary Wall Biosynthesis and Novel Regulators for Vascular Cambium Development in Populus
Source: Genes (Basel). 2019 Sep 7;10(9):690. doi: 10.3390/genes10090690 (PMC6770981; doi:10.3390/genes10090690)
Supplement: Supplementary file 1 [file genes-10-00690-s001.zip › genes-588507/Figure S1 and S2.docx]

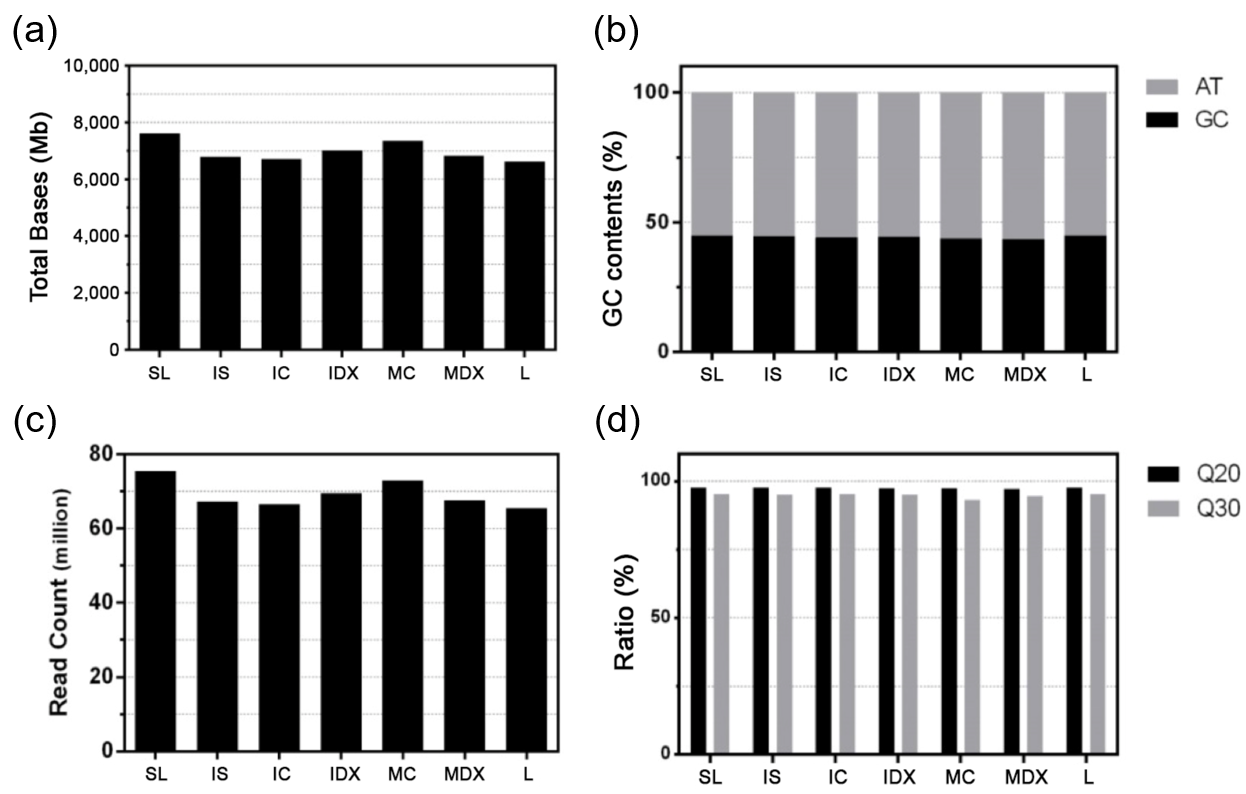


**Figure S1.** RNA-seq raw data processing. Results of RNA-seq raw data processing per RNA-seq libraries. (**a**) Total number of bases in the sequence reads identified in this analysis. (**b**) AT and GC content of the sequence reads. (**c**) Total number of the sequence reads. (**d**) Quality of the sequence reads; per cent ratio of phred score above 20 (i.e., Q20) and 30 (i.e., Q30) was shown.


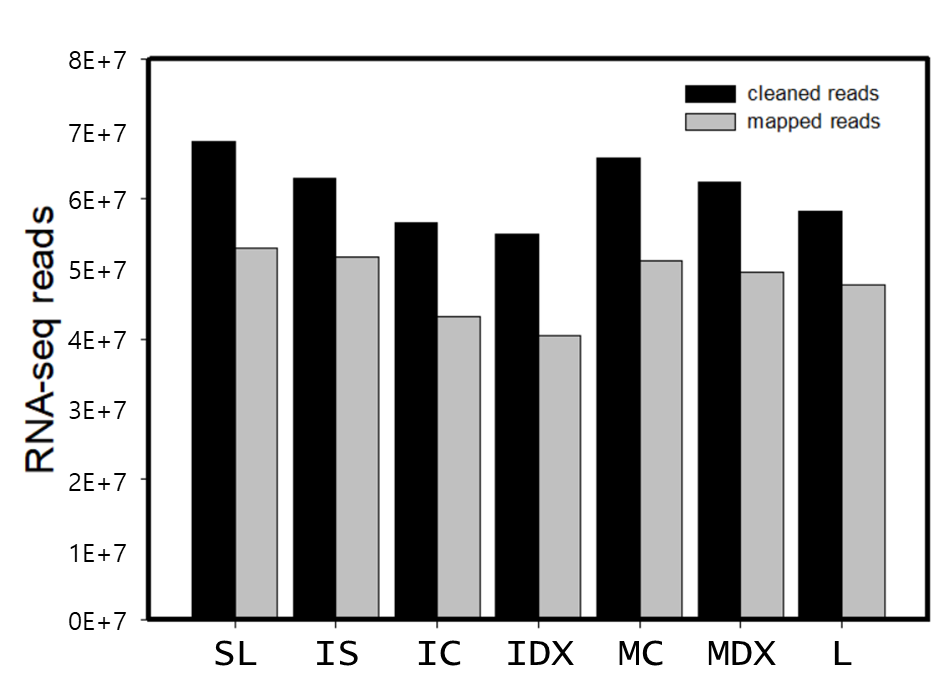


**Figure S2.** Mapping of the sequence reads to the genome of *Populus trichocarpa.* Number of cleaned-up sequence reads from each RNA-seq library (black) was mapped to the *P. trichocarpa* genome (v3.0). Resulting number of mapped reads are shown in gray.


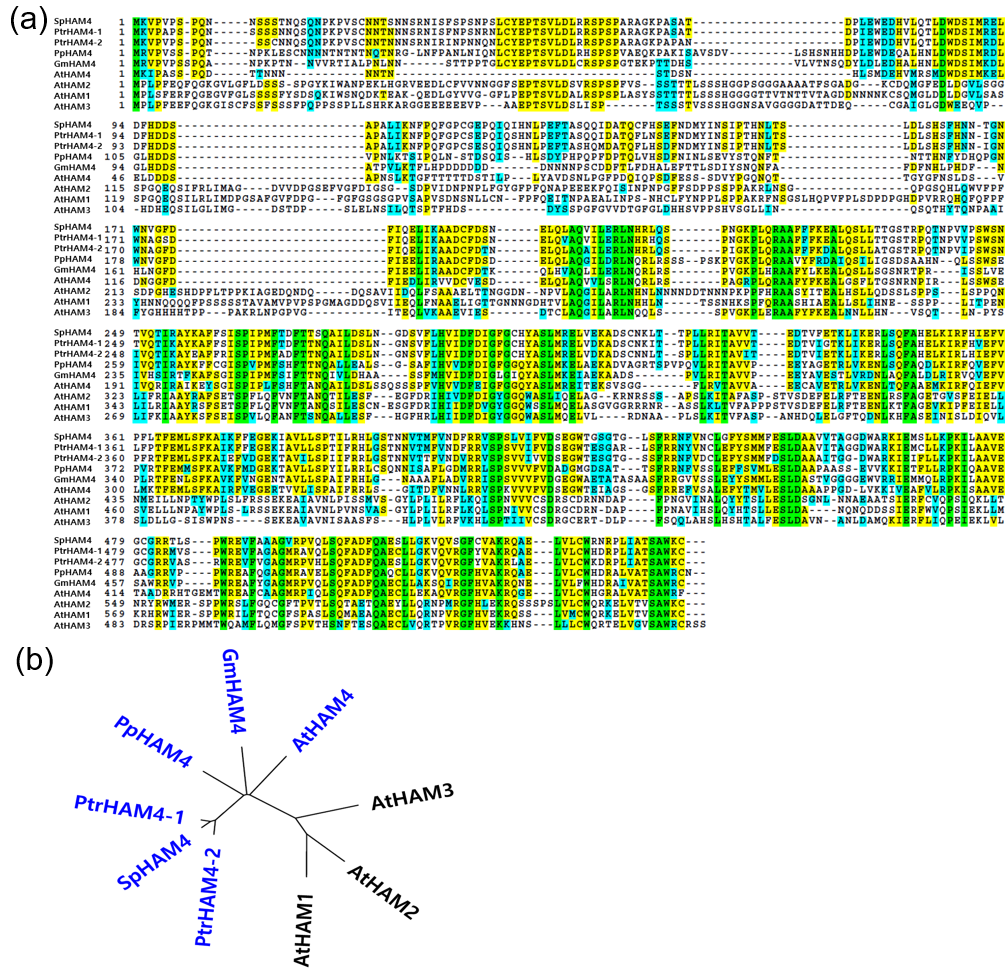


**Figure S3.** *PtrHAM4-1* is homologous to *AtHAM4.* (**a**) Amino acid sequence alignment of the *AtHAM* family with *AtHAM4* homologous genes from other plant species. Gene name and locus IDs are as follows: *PtrHAM4-1* (*Populus trichocarpa*, Potri.005G125800.1); *PtrHAM4-2* (Potri.007G029200.1); *PpHAM4* (*Prunus persica*, Prupe.7G150900.1); *GmHAM4* (*Glycine max*, Glyma.01G177200.1); *AtHAM4* (*Arabidopsis thaliana*, AT4G36710.1); *AtHAM2* (*A. thaliana*, AT3G60630.1); *AtHAM1* (*A. thaliana*, AT2G45160.1); and *AtHAM3* (*A. thaliana*, AT4G00150.1). (**b**) Phylogenetic analysis of genes aligned in (a). Complete amino acid sequences were aligned using ClustalW and the non-rooted tree was constructed using MEGA 6.0 [1] with a bootstrap value 1,000.

References

1. Tamura, K.; Stecher, G.; Peterson, D.; Lilipski, A.; Kumar, S. MEGA6: Molecular evolutionary genetics analysis version 6.0. Mol. Biol. Evolut. 2013, 30, 2725–2729.
